# Integrated Optofluidic Chip for Low-Volume Fluid Viscosity Measurement

**DOI:** 10.3390/mi8030065

**Published:** 2017-02-23

**Authors:** Tie Yang, Giovanni Nava, Valerio Vitali, Francesca Bragheri, Roberto Osellame, Tommaso Bellini, Ilaria Cristiani, Paolo Minzioni

**Affiliations:** 1 Department of Electrical, Computer, and Biomedical Engineering, Università di Pavia, Via Ferrata 5A, 27100 Pavia, Italy; yangtie@gmail.com (T.Y.); valerio.vitali01@universitadipavia.it (V.V.); ilaria.cristiani@unipv.it (I.C.); 2 Department of Biomedical Science and Translational Medicine, Università di Milano, Via F.lli Cervi 91, 20090 Segrate, Italy; giovanni.nava@unimi.it (G.N.); tommaso.bellini@unimi.it (T.B.); 3 Institute of Photonics and Nanotechnology, CNR & Department of Physics, Politecnico di Milano, Piazza Leonardo da Vinci 32, 20133 Milano, Italy; francesca.bragheri@ifn.cnr.it (F.B.); roberto.osellame@polimi.it (R.O.)

**Keywords:** optofluidics, viscosity, microfluidics, optical forces, optical shooting

## Abstract

In the present work, an integrated optofluidic chip for fluid viscosity measurements in the range from 1 mPa·s to 100 mPa·s is proposed. The device allows the use of small sample volumes (<1 µL) and the measurement of viscosity as a function of temperature. Thanks to the precise control of the force exerted on dielectric spheres by optical beams, the viscosity of fluids is assessed by comparing the experimentally observed movement of dielectric beads produced by the optical forces with that expected by numerical calculations. The chip and the developed technique are validated by analyzing several fluids, such as Milli-Q water, ethanol and water–glycerol mixtures. The results show a good agreement between the experimental values and those reported in the literature. The extremely reduced volume of the sample required and the high flexibility of this technique make it a good candidate for measuring a wide range of viscosity values as well as for the analysis of nonlinear viscosity in complex fluids.

## 1. Introduction

Microrheology recently emerged among the techniques used to characterize the mechanical properties of soft materials or fluids, thanks to the possibility to study the response of samples at the molecular level. In addition, the capability to use small sample volumes (few µL or even less) also opened the way to the application of these techniques to expensive/limited materials, such as DNA-based samples [1,2]. The rheological properties of a sample can generally be studied at the micro-scale level by exploiting the thermal motion of micro-probes inserted into the sample, thus scanning the mechanical response in the linear regime [3,4]. However, the possibility to force the probe movements opens the way both to the measurement of high-viscosity samples, where the detection of the Brownian motion of the probe could be extremely challenging, and to the study of the material response to strong and impulsive stresses. Such techniques generally apply optical or magnetic forces to a probe (or a multitude of probes) inside the material without affecting it directly [5,6,7]. In particular, optical forces are a natural candidate for microrheology, as they can be finely controlled and easily integrated in microfluidic devices.

In the present work, we present a glass-based microfluidic chip suitable for measuring the viscosity of a fluid even with a sample volume <1 µL. This is achieved thanks to the force applied by two optical beams, propagating through the chip microchannel, on microbeads inserted in the sample and used as a probe. This force permits a precise micro-manipulation of the bead, allowing the study of its motion and hence the extraction of the fluid viscosity.

The complete experimental procedure implemented to collect the data is explained in Section 3. The optical-force profile characterization is described in Section 4.1 and the obtained values have been used to perform the measurement on various glycerol–water mixtures as well as on water and on ethanol (Section 4.2 and Section 4.3). The reported results show the capability of the optofluidic micro-rheological setup to measure viscosity values between 1 mPa·s and 100 mPa·s and also to explore the temperature dependence of fluid viscosity in the range 20–40 ℃.

## 2. Device Structure and Working Principle

### 2.1. Chip Design and Fabrication

**Figure 1:**
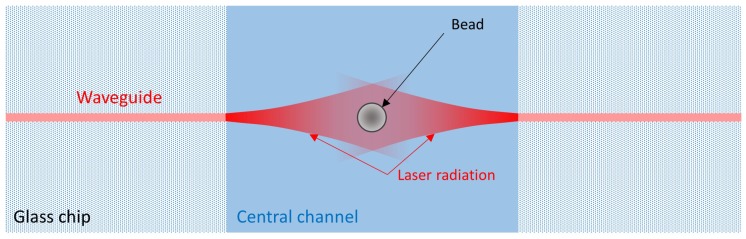
Schematic structure of an optical stretcher: a single bead is trapped between two facing non-focused Gaussian laser beams, with the same optical power, output by two integrated waveguides.

Optical forces provide a contactless and precise way to manipulate microbeads suspended in a fluid; hence optical tweezers have already been used to measure complex fluid viscosity as reported in [1,8,9]. In this study, we realize a simple micro-rheometer by using a different approach that exploits the forces available in an integrated dual-beam laser trap [10,11,12,13,14]. The device structure is schematically shown in Figure 1. The optofluidic chip used for the experiments is fabricated by directly integrating optical waveguides through femtosecond laser writing in a commercial microfluidic chip (Translume Inc., Ann Arbor, MI, USA). Details about the chip fabrication and the laser writing technique can be found in [13,15,16]. The finished device has a single straight channel with a square section of 150 µm × 150 µm, and a pair of facing waveguides on the two sides of the channel at half of the channel height (i.e., 75 µm from the channel floor). The counter-propagating beams from the two facing waveguides apply two forces on the microbead, thus allowing to stably trap it when the two laser beams exhibit the same optical power, as depicted in Figure 1.

The trapping capability can be used to capture a single microbead flowing in the channel, but the optical beams also allow the movement of the bead across the transverse dimension of the microchannel by unbalancing the power of one of the two beams, thus shifting the equilibrium-position of the bead along the beam propagation axis.

### 2.2. Fluid Viscosity Derivation

The forces exerted on the microbead by the laser beams have already been studied in detail elsewhere [17,18]. As non-focused Gaussian laser beams are considered and the bead diameter is comparable with the laser beam waist, the paraxial ray optics approach is adopted, as reported in the literature [13,19,20].

Once the bead is illuminated by the two laser beams, the gradient force automatically aligns the particle with the beam axis, where the gradient force is zero, and only the optical scattering force is present. Thus, only the scattering component of the optical force ($F_{S}$) causes the bead movement until the equilibrium position is reached. To correctly evaluate the optical force applied on a dielectric sphere by a light beam, it is necessary to decompose the light beam into a series of optical rays, to separately calculate the force applied by each ray ($F_{1ray}$) on the sphere due to the photon momentum variation and then to sum up all the force contributions. This calculation, which is described in detail in many scientific papers [19,21,22], is based on the idea that each optical ray will apply a scattering force (i.e., a force pushing the particle along the beam propagation axis) which depends on the refractive index of the medium ($n_{m}$), on the power (*P*) associated to the optical ray, on the reflection coefficient at the medium–particle interface (*R*) and on the incidence and transmittance angles (θ,γ) formed by the ray while crossing the particle surface.

(1) $$F_{S,1ray}=\frac{n_{m}P}{c}\times \left\{\left[1+R\cos\left(2\theta \right)\right]-\frac{T^{2}\left[\cos\left(2\theta -2\gamma \right)+R\cos\left(2\theta \right)\right]}{1+R^{2}+2R\cos\left(2\gamma \right)}\right\}$$

When the bead moves inside the fluid, pushed by the scattering force, it also experiences the Stokes drag force, which slows down its movement. The drag force $F_{d}$ is related to the fluid viscosity and can be analytically reported as follows

(2) $$F_{d}=6\pi \eta a\dot{x}/\gamma$$

(3) $$\gamma \left(x\right)=1-\frac{9}{32}\frac{D}{x}+\frac{1}{8}{\left(\frac{D}{2x}\right)}^{3}-\frac{45}{256}{\left(\frac{D}{2x}\right)}^{4}-\frac{1}{16}{\left(\frac{D}{2x}\right)}^{5}$$

where *η* is the fluid viscosity, $\dot{x}$ is the velocity of the bead, *a* and *D* are the radius and diameter of the bead respectively and *γ* is the correction parameter to be necessarily considered when the bead is close to one of the microchannel surfaces [23].

As the inertial effects during the movement of the particle inside the fluid can be neglected, because of the extremely low Reynolds number (<10${}^{-3}$), it is immediate to derive that in each position the total optical force, $F_{S,TOT}\left(x\right)$ due to the sum of the contribution from all rays, must be equal to the drag force, see Equation (4), and thus the bead moves with a position-dependent velocity that is equal to the speed-limit in that medium

(4) $$F_{S,TOT}\left(x\right)=F_{d}\left(\dot{x},x\right)$$

Thanks to the fact that the scattering force profile $F_{S,TOT}\left(x\right)$ can be calculated numerically with good precision, it is also possible to derive the curve of the bead position as a function of time, which will be indicated as “bead trajectory” in the following. By comparing the experimentally retrieved bead trajectory with the theoretically calculated ones, the fluid viscosity can thus be assessed.

## 3. Experiment and Methods

### 3.1. Experimental Setup

**Figure 2:**
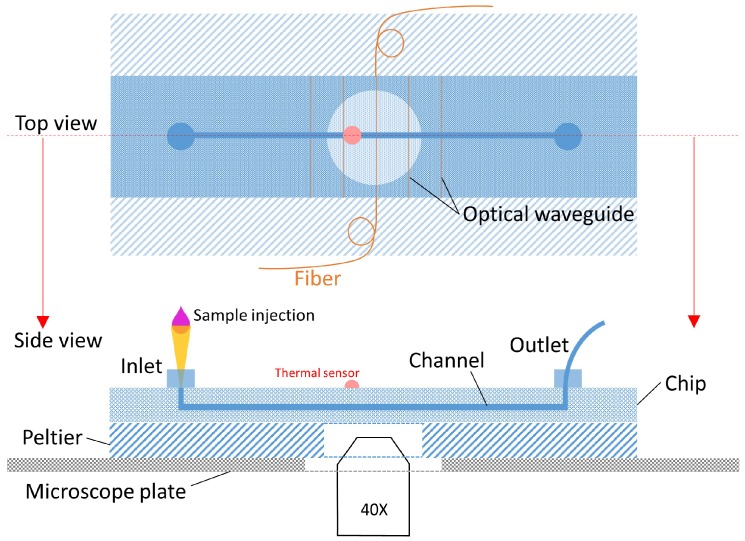
Top and side views of the experimental setup configuration. The low volume sample is injected by a micropipette into the microchannel of the chip.

A schematic of the experimental setup, which is positioned on a phase-contrast inverted microscope, is shown in Figure 2, with the tubings of a computer-controlled micropump already connected to one of the two Luer connectors present on the surface of the microfluidic chip. The sample is prepared by mixing, in a vial, a very small amount of poly(methyl methacrylate) (PMMA) microbeads (10 µm diameter) into the target fluid. A standard micro-pipette (not shown in the figure) is used to inject the sample into the microfluidic chip reservoir. Sample movement in the channel is governed by capillarity, but it can be easily controlled by the micropump, allowing the sample to be pushed in the section of the microchannel where the laser radiation is present. The setup is equipped with a temperature control system, which consists of a small Peltier module placed below the chip and a thermal sensor attached on top of the chip, which produces the feedback signal for the Peltier control. Thanks to a proper calibration, this system allowed the obtainment of a temperature accuracy of ±0.2 ℃. Two single-mode optical fibers are used to couple the radiation emitted by an Yb-doped fiber laser (emission wavelength 1070 nm) to the optical waveguides integrated in the chip, and two U-benches are inserted along the optical paths to allow an easy control of the optical power in the two branches. Thanks to a custom LabVIEW program (realized using LabVIEW 2012, by National Instruments, Austin, TX, USA), the entire system, including a charge coupled device (CCD) camera connected to the microscope, is controlled by using a simple computer interface.

### 3.2. Optical Shooting Protocol and Data Acquisition

**Figure 3:**
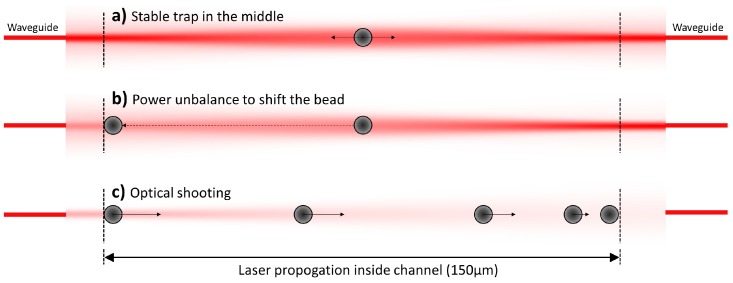
Optical shooting experimental protocol. (**a**) A single bead is captured by two laser beams with equal power; (**b**) the bead is shifted to the left side by decreasing the optical power emitted from the left waveguide; (**c**) once the bead is close to the left channel border, the opposite laser beam is abruptly blocked and then the optical shooting procedure is performed, i.e., the microbead is moved only by the left laser across the whole channel and the movement is recorded by a charge coupled device (CCD) camera.

After the sample is injected into the microfluidic channel, the pressure is adjusted to produce a suitable flowing speed of the fluid. Once a bead is captured by the laser beams, the flow is stopped. The trapped bead is then stabilized in the middle of the channel, see Figure 3a. The laser power from the left side is then decreased by increasing the optical attenuation in the corresponding U-bench, and the bead consequently shifts to the left side as shown in Figure 3b. When the bead is close to the left border of the channel, the right laser beam is abruptly blocked and the bead shooting is recorded by a CCD camera, Figure 3c. The optical shooting procedure is repeated several times with the same bead, then the bead is released, the pressure is re-increased to control the sample flow and the procedure is repeated.

It is important to highlight that the optical attenuator inserted along the optical path has a known and constant attenuation factor, so that the shooting power is always the same unless the power source is changed.

**Figure 4:**
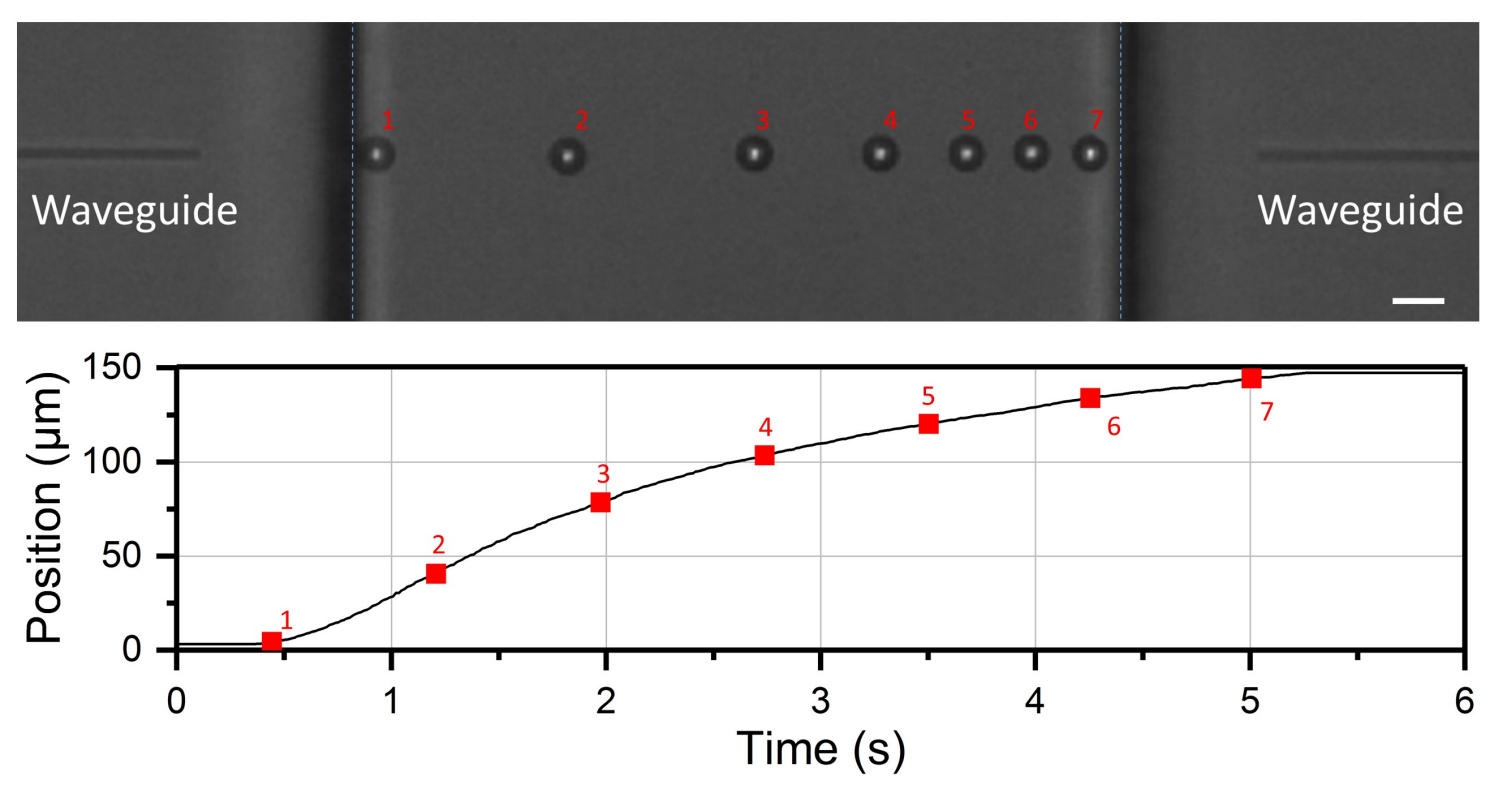
Phase-contrast microscope image stack showing the sequential microbead positions at equal time intervals from an optical shooting experiment. Scale bar: 10 µm. The bright spot in the bead center is used to retrieve the bead position. The extracted shooting trajectory is shown underneath. The red numbers in the two figure panels correspond to the same bead position.

During the bead optical shooting experiment, the process is monitored by a CCD camera with high frame rate. Thanks to the phase contrast mode, the image of the microbead has a very bright spot in the center, which makes it possible to identify the bead position with high accuracy. The LabVIEW software used for the setup management allows also for real-time extraction of the bead trajectory from the bead shooting experiment, as shown in Figure 4. The stacked microscope images show the positions sequentially occupied by the bead after equal time intervals, as indicated by the numbers, and the corresponding displacement is reported underneath. An interesting advantage offered by the real-time analysis approach is that it allows the evaluation of whether any problem (e.g., unstable laser power or residual fluid flow) is affecting the measurement, so that solutions can be immediately found, without having to carry out all the measurements and all the analysis before noticing any possible issue.

## 4. Results and Discussion

### 4.1. Optical Force Profile and Theoretical Trajectory

Standard PMMA microbeads, with a 10 µm diameter and a refractive index of 1.482 (@ 1070 nm) were introduced in the different fluids included in this study: Milli-Q water, ethanol, and water–glycerol mixtures.

**Figure 5:**
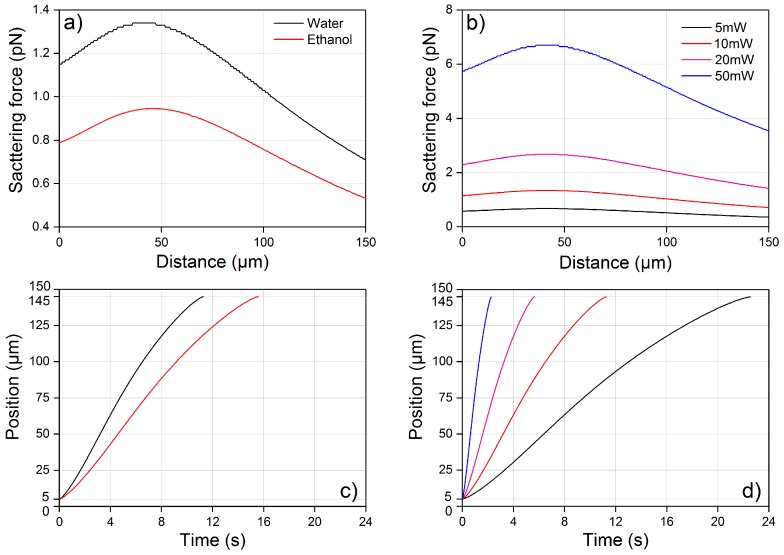
Theoretical calculations of the $F_{S,TOT}$ profile exerted on a poly(methyl methacrylate) (PMMA) bead of 10 µm diameter and of its corresponding optical shooting trajectory from the left laser beam only. (**a**) $F_{S,TOT}$ along the laser beam axis, and across the full channel width, is calculated for both water and ethanol at the same laser power of 10 mW; (**b**) $F_{S,TOT}$ in water, calculated for different laser powers; (**c**,**d**) are the optical shooting trajectories corresponding to the optical force profile in (**a**,**b**), respectively.

Firstly, it is necessary to calculate the optical force profile to allow the fitting of the experimental bead-displacement curves with the theoretical ones. Thanks to the automatic centering of the bead along the beam axis, only the scattering force along the laser beam axis is responsible for the bead shooting and the force calculation is quite simple. The beam waist in our chip was previously characterized to be 3.8 µm [24]. In Figure 5a, we report the force ($F_{S,TOT}$) applied across the channel by a 10 mW laser beam on a PMMA microbead suspended in water and in ethanol, with a refractive index of 1.325 and 1.354 respectively. As expected by Equation (1), a change of the refractive index of the fluid significantly impacts the force magnitude, and the forces applied to the bead increase when the refractive index difference between the bead and the fluid is increased. This can be intuitively understood by considering that a larger refractive index difference causes a larger momentum change of photons at the interface, thus resulting in a larger optical force. It can also be observed that the force profiles in water and ethanol have similar shapes: the force initially increases, reaches a maximum value and then continuously decreases, and only a slight shift in the maximum-force position, due to a change in the beam divergence [18,19,21], can be observed. In Figure 5b, we show the different $F_{S,TOT}$ profiles calculated for different values of the optical power, considering water as a fluid. As the $F_{S,TOT}$ is obtained by summing up the different $F_{S,1ray}$ contributions, which are directly proportional to *P* (see Equation (1)), all the profiles are simply scaled copies of the same curve.

Starting from the optical force profile, the correspondent bead trajectory can be calculated as discussed in Section 2.2. In Figure 5c,d, we show the calculated bead trajectories corresponding to the $F_{S,TOT}$ reported in Figure 5a,b respectively. It should be noted that the starting and finish positions are set to 5 µm and 145 µm (instead of 0 µm and 150 µm) as the bead radius is equal to 5 µm.

It is important to highlight that all the trajectories reported in Figure 5d, obtained considering different *P* values, can be overlapped by simply re-scaling the time axis by a factor corresponding to the considered optical power. This consideration, which may appear trivial, is of significant importance because it allows to test for the presence/absence of viscosity changes induced by the laser-heating effect. In fact, in the case of constant viscosity, the experimental shooting-trajectories curves obtained at different power levels must overlap once the time axis is properly re-scaled, while a deviation should be expected in the case of a laser-heating induced viscosity change [25]. Similarly, the system linearity can be verified by plotting the curves with a normalized position and time: in this case, all the curves—independently of the medium viscosity and of the laser power—collapse onto a “master curve” whose shape depends only on the optical force profile across the channel.

**Figure 6:**
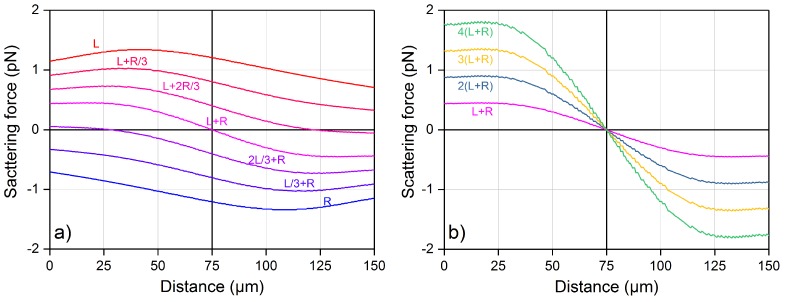
Total scattering force, as a function of the position inside the microchannel, produced by two facing laser beams on a PMMA bead of 10 µm diameter suspended in water. The laser power values are varied by multiplying 10 mW times the factor indicated by the curve labels. Capital letter “L” stands for the left laser beam and “R” for the right one.

Starting from the same conditions as in Figure 5b, i.e., 10 µm PMMA beads suspended in water, the $F_{S,TOT}$ profile produced by two facing laser beams is further investigated. Different laser powers from the two laser beams are studied and the result is reported in Figure 6. In panel (a), we show the effect of unbalanced power between the two beams, and we use the labels next to the force curves to indicate the factor multiplying the nominal power from either the left (L), or the right (R) laser. When the force is positive, the bead will be pushed to the right direction and vice-versa. On the other hand, if we consider two conter-propagating laser beams with the same power, see Figure 6b, the force profiles always cross the zero line, with negative derivative, exactly in the middle of the channel, showing that it is a stable trapping point, corresponding to the bead-trapping situation depicted in Figure 3a. For the optical shooting experiment, the microbead is shifted to the left channel border by introducing a carefully selected attenuator in the left-branch U-bench, as shown in Figure 3b, so that the bead shifts to the new trapping position without making contact with the channel wall. This procedure allows obtaining a precise and repeatable starting position of all the bead trajectories, even when the laser power *P* is changed, as the trapping position is not influenced by the absolute value of *P*, but only by the ratio of the power from the two sides.

### 4.2. Viscosity of Water and Ethanol as a Function of Temperature

The viscosity of Milli-Q water was initially studied to verify the method accuracy. Measurements were performed at five different temperatures: 20 ℃, 25 ℃, 30 ℃, 35 ℃ and 40 ℃. For each temperature, the optical shooting experiment was repeated several times with the same bead, and then repeated with new beads. As water has a relatively low viscosity value, the shooting laser power was set to a very low value, and the absence of laser-heating effect was proven by trajectory shifting at different laser powers, as previously discussed. The measurements at different temperatures were initially performed while increasing the temperature from 20 ℃ to 40 ℃ by 5 ℃ steps and then repeated while decreasing the temperature, to check for measurements’ reliability and reproducibility. When the temperature is increased, the water viscosity decreases and thus the trajectories show a faster bead movement.

**Figure 7:**
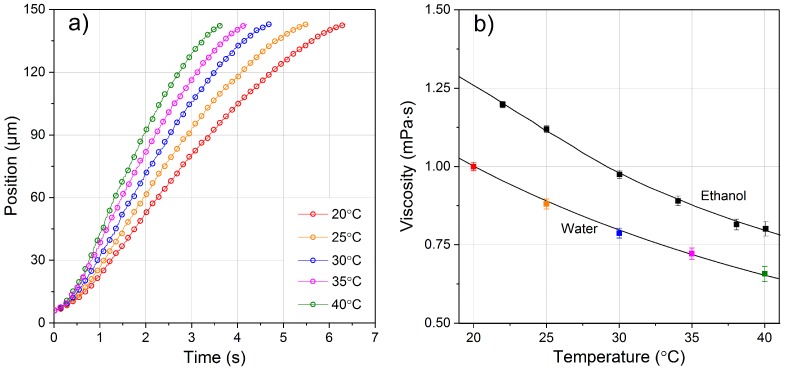
(**a**) Experimentally retrieved optical shooting trajectories of a PMMA bead in water at the same optical power but at different temperature values. The error bars of the position would be smaller than the circle size itself and are thus not shown. Note that all the trajectories have the same starting and finishing positions; (**b**) water and ethanol viscosity values at different temperatures are retrieved from the shooting trajectories. Viscosity for water corresponds to the trajectories in (**a**). Obtained results in dots are compared with curves reported in literature [26,27].

The extracted optical shooting trajectories and the obtained viscosity are reported in Figure 7. It can be seen that all the shooting trajectories have the same starting and finishing positions as they have the same stable trapping position near the left channel border and the same movement limitation because of the right channel border. Besides, the trajectories show only very small variations between each measurement at the same condition, as can be seen by the error bars in Figure 7b. In Figure 7b, the retrieved viscosity values of both water and ethanol are compared with data reported in literature [26,27] and they show an excellent match between experimental and theoretical data. It is interesting to notice that at high temperatures the viscosity has a slightly larger error bar, due to the stronger Brownian motion observed during the shooting experiments. The obtained results confirm that the proposed protocol of optical shooting measurement provides an efficient method to evaluate the fluid viscosity. Other temperature values can be easily achieved by expanding the applied temperature range.

### 4.3. Glycerol Viscosity Measurement

Given the good results obtained on testing water and ethanol viscosity, we moved to test a more viscous fluid, as glycerol. Pure glycerol is in fact much more viscous than water (about 1000 times higher at room temperature), and its viscosity varies significantly with temperature. We thus created mixtures of glycerol with Milli-Q water, at different mass concentration, and we studied them at different temperatures.

**Figure 8:**
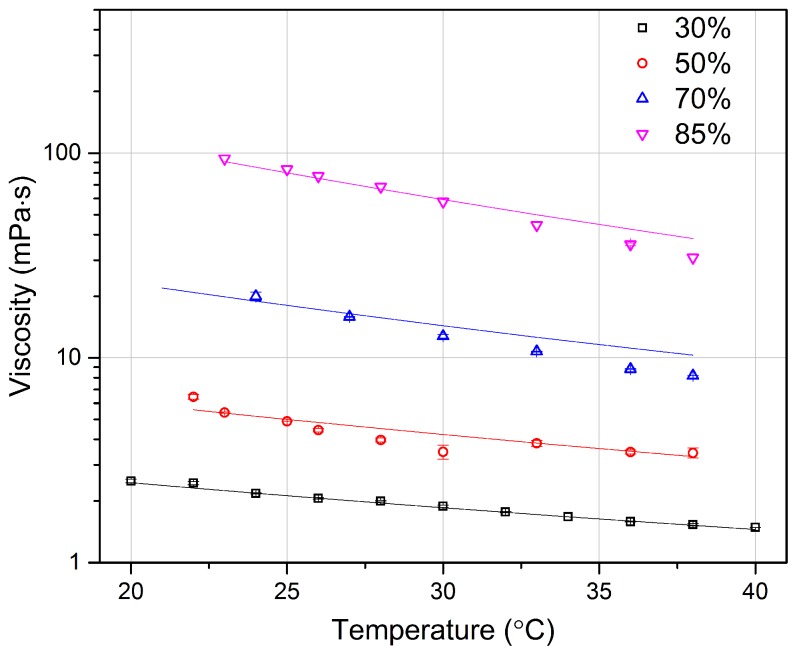
Viscosity of glycerol mixtures with water at different mass concentrations and different temperatures. Experimental data in scatter symbols are compared with results from literature [28]. Because of the larger variation of viscosity at different concentrations, the logarithmic scale is selected to have a better visualization of the results.

The obtained viscosity values, which are displayed in Figure 8, highlight that at low glycerol concentration, our results are in good agreement with data from the literature. At high glycerol concentrations (70% and 85%), a small deviation between our data and those reported in the literature can be observed. We speculate that this can be due to a non-perfect mixing of the two fluids, even with multiple cycles of vortex mixing and supersonic vibration.

With the same temperature change, the glycerol mixture at higher concentration shows much larger variation, especially for 70% and 85% concentration. It should be mentioned that at higher concentration of glycerol, a higher laser power is required to have a full trajectory along the whole channel width compared to the lower concentration cases. Moreover, to avoid the laser heating effect when the fluid temperature is changed and consequent viscosity variation in the area near the waveguide, the initial part of the trajectory (about 20 µm) is discarded; this does not affect the viscosity retrieval process because of the non-inertial behavior in microfluidic condition, as stated in Section 2.2.

## 5. Conclusions

In conclusion, an integrated optofluidic chip is presented in this work that allows the measurement of fluid viscosity, and very low fluid sample consumption is achieved by using micropipette injection. By exploiting optical forces, the chip allows microbeads inside a fluid to be precisely controlled and manipulated, giving the possibility to assess fluid viscosity. The chip is also equipped with a temperature control, to maintain the fluid at the selected temperature. The fluid viscosity is derived by fitting the microbead optical shooting trajectory with theoretical curves. By real-time analysis, the shooting measurement can be repeated with the same bead in the same position of the fluid and with different beads in different positions of the sample, offering very high reliability and consistency. The chip has been successfully applied to measure the viscosity of several fluids and the obtained results well match the data already reported in the scientific literature. Additionally, the measurement range of fluid viscosity demonstrated in this study is very large thanks to the simple optical force control permitted by a simple variation of the laser power. However, higher laser power may introduce a laser heating effect and this can be evaluated and avoided by shooting trajectory shifting and partial trimming.

The proposed chip and the technique reported here can also be directly applied to test the viscosity of fluids exhibiting more complex mechanical behaviors, as in the case of non-Newtonian fluids. It is interesting to notice that if non-Newtonian fluids are considered, a non-linear relationship between $F_{S,TOT}$ and $\dot{x}$ should be observed, thus yielding a change in the shape of the bead trajectories. For this reason, it could be interesting in this case to use an approach based on normalized position and time parameters, as proposed in a recent paper, where optical forces are exploited with a different approach to measure the viscosity of samples [8].

## References

[1] Chapman C.D., Robertson-Anderson R.M. (2014). Nonlinear microrheology reveals entanglement-driven molecular-level viscoelasticity of concentrated DNA. Phys. Rev. Lett..

[2] Gardel M.L., Valentine M.T., Crocker J.C., Bausch A.R., Weitz D.A. (2003). Microrheology of entangled F-actin solutions. Phys. Rev. Lett..

[3] Squires T.M., Mason T.G. (2010). Fluid mechanics of microrheology. Ann. Rev. Fluid Mech..

[4] Mason T.G., Weitz D.A. (1995). Linear viscoelasticity of colloidal hard sphere suspensions near the glass transition. Phys. Rev. Lett..

[5] Squires T.M. (2005). Active microrheology in the continuum limit: Can the macrorheology be recovered?. Differences.

[6] Tassieri M., Gibson G.M., Evans R.M.L., Yao A.M., Warren R., Padgett M.J., Cooper J.M. (2010). Measuring storage and loss moduli using optical tweezers: Broadband microrheology. Phys. Rev. E.

[7] Atakhorrami M., Sulkowska J.I., Addas K.M., Koenderink G.H., Tang J.X., Levine A.J., MacKintosh F.C., Schmidt C.F. (2006). Correlated fluctuations of microparticles in viscoelastic solutions: Quantitative measurement of material properties by microrheology in the presence of optical traps. Phys. Rev. E.

[8] Tassieri M., Giudice F.D., Robertson E.J., Jain N., Fries B., Wilson R., Glidle A., Greco F., Netti P.A., Maffettone P.L., Bicanic T., Cooper J.M. (2015). Microrheology with optical tweezers: Measuring the relative viscosity of solutions ‘at a glance’. Sci. Rep..

[9] Keen S., Yao A., Leach J., Di Leonardo R., Saunter C., Love G., Cooper J., Padgett M. (2009). Multipoint viscosity measurements in microfluidic channels using optical tweezers. Lab Chip.

[10] Bellini N., Vishnubhatla K.C., Bragheri F., Ferrara L., Minzioni P., Ramponi R., Cristiani I., Osellame R. (2010). Femtosecond laser fabricated monolithic chip for optical trapping and stretching of single cells. Opt. Express.

[11] Bragheri F., Minzioni P., Martinez Vazquez R., Bellini N., Paie P., Mondello C., Ramponi R., Cristiani I., Osellame R. (2012). Optofluidic integrated cell sorter fabricated by femtosecond lasers. Lab Chip.

[12] Zhang Y., Watts B., Guo T., Zhang Z., Xu C., Fang Q. (2016). Optofluidic device based microflow cytometers for particle/cell detection: A review. Micromachines.

[13] Yang T., Bragheri F., Minzioni P. (2016). A comprehensive review of optical stretcher for cell mechanical characterization at single-cell level. Micromachines.

[14] Matteucci M., Triches M., Nava G., Kristensen A., Pollard M., Berg-Sørensen K., Taboryski R. (2015). Fiber-based, injection-molded optofluidic systems: Improvements in assembly and applications. Micromachines.

[15] Yang T., Paiè P., Nava G., Bragheri F., Vazquez R.M., Minzioni P., Veglione M., Di Tano M., Mondello C., Osellame R. (2015). An integrated optofluidic device for single-cell sorting driven by mechanical properties. Lab Chip.

[16] Nava G., Bragheri F., Yang T., Minzioni P., Osellame R., Cristiani I., Berg-Sørensen K. (2015). All-silica microfluidic optical stretcher with acoustophoretic prefocusing. Microfluid. Nanofluid..

[17] Ashkin A. (1970). Acceleration and trapping of particles by radiation pressure. Phys. Rev. Lett..

[18] Ashkin A. (1978). Trapping of atoms by resonance radiation pressure. Phys. Rev. Lett..

[19] Ferrara L., Baldini E., Minzioni P., Bragheri F., Liberale C., Fabrizio E.D., Cristiani I. (2011). Experimental study of the optical forces exerted by a Gaussian beam within the Rayleigh range. J. Opt..

[20] Bragheri F., Ferrara L., Bellini N., Vishnubhatla K.C., Minzioni P., Ramponi R., Osellame R., Cristiani I. (2010). Optofluidic chip for single cell trapping and stretching fabricated by a femtosecond laser. J. Biophotonics.

[21] Ashkin A. (1992). Forces of a single-beam gradient laser trap on a dielectric sphere in the ray optics regime. Biophys. J..

[22] Minzioni P., Bragheri F., Liberale C., Di Fabrizio E., Cristiani I. (2008). A novel approach to fiber-optic tweezers: Numerical analysis of the trapping efficiency. IEEE J. Select. Top. Quantum Electron..

[23] Svoboda K., Block S.M. (1994). Biological applications of optical forces. Annu. Rev. Biophys. Biomol. Struct..

[24] Yang R., Li R. (2016). Optical force exerted on a Rayleigh particle by a vector arbitrary-order Bessel beam. J. Quant. Spectrosc. Radiat. Transf..

[25] Ebert S., Travis K., Lincoln B., Guck J. (2007). Fluorescence ratio thermometry in a microfluidic dual-beam laser trap. Opt. Express.

[26] Thermophysical Properties of Water and Steam International Association for the Properties of Water and Steam. http://www.viscopedia.com/viscosity-tables/substances/water/.

[27] Tables of Physical and Chemical Constants Kaye and Laby Online. http://www.viscopedia.com/viscosity-tables/substances/ethanol/.

[28] Cheng N.S. (2008). Formula for the viscosity of a glycerol–water mixture. Ind. Eng. Chem. Res..

